# Pseudo-Mannosylated DC-SIGN Ligands as Potential Adjuvants for HIV Vaccines

**DOI:** 10.3390/v6020391

**Published:** 2014-01-27

**Authors:** Angela Berzi, Norbert Varga, Sara Sattin, Patrizio Antonazzo, Mara Biasin, Irene Cetin, Daria Trabattoni, Anna Bernardi, Mario Clerici

**Affiliations:** 1 Department of Biomedical and Clinical Sciences “L. Sacco”, University of Milan, Via GB. Grassi 74, 20157 Milan, Italy; E-Mails: mara.biasin@unimi.it (M.B.); daria.trabattoni@unimi.it (D.T.); 2 Department of Chemistry, University of Milan, Via C.Golgi 19, 20133 Milan, Italy; E-Mails: norbert.varga@unibas.ch (N.V.); sara.sattin@unimi.it (S.S.); anna.bernardi@unimi.it (A.B.); 3 Unit of Obstetrics and Gynecology, Department of Biomedical and Clinical Sciences “L. Sacco”, University of Milan, Via GB. Grassi 74, 20157 Milan, Italy; E-Mails: Antonazzo.Patrizio@hsacco.it (P.A.); irene.cetin@unimi.it (I.C.); 4 Department of Pathophysiology and Transplantation, University of Milan, Via F.lli VCervi 93, 20090 Milan, Italy; E-Mail: mario.clerici@unimi.it; 5 Don C. Gnocchi Foundation, IRCCS, Via Capecelatro 66, 20148 Milan, Italy

**Keywords:** vaccine, HIV-1, adjuvant, DC-SIGN, innate immunity, glycomimetic compounds

## Abstract

The development of new and effective adjuvants may play a fundamental role in improving HIV vaccine efficacy. New classes of vaccine adjuvants activate innate immunity receptors, notably toll like receptors (TLRs). Adjuvants targeting the C-Type lectin receptor DC-SIGN may be alternative or complementary to adjuvants based on TRL activation. Herein we evaluate the ability of the glycomimetic DC-SIGN ligand Polyman 19 (**PM 19**) to modulate innate immune responses. Results showed that **PM 19** alone, or in combination with TLR agonists, induces the expression of cytokines, β chemokines and co-stimulatory molecules that may, in turn, modulate adaptive immunity and exert anti-viral effects. These results indicate that the suitability of this compound as a vaccine adjuvant should be further evaluated.

## 1. Introduction

Several efforts are needed to develop an effective and safe vaccine against AIDS. The use of live attenuated viruses is not feasible in humans for safety concerns. On the other hand, vaccines based on protein subunits are scarcely immunogenic. Other approaches are based on DNA vaccines or live recombinant vectors encoding HIV-1 antigens to enhance cell mediated immunity (CMI). Prime-boost strategies, that use a combination of different types of vaccines to generate strong CMI and humoral immune responses, have been developed [1]. However, clinical trials based on this approach have not so far produced the desired results. Thus, the prime-boost combination of vaccines used in the RV144 trial demonstrated only a limited preventative effect [2]. Even more recently, in April 2013, the HVTN 505 vaccine clinical trial was halted because the regimen failed to prevent HIV infection, nor even to decrease viral load among the vaccinees that became infected by the virus during the trial [3]. 

However, alum is a relatively poor adjuvant and, in particular, its effects on the induction of cytotoxic T-lymphocytes (CTLs) are marginal at best [4]. The development of novel, more potent adjuvants that could induce virus-specific Th1 and CTL responses may improve HIV-1 vaccine efficacy.

Adjuvants used to be developed in an empirical manner, without knowing completely their mode of action, but it is now becoming clear that most adjuvants exert their effects upon interacting with the innate immune system. Indeed activation of the innate immunity plays a fundamental role in initiating and shaping the adaptive immune response. Pattern recognition receptors (PPRs) are attractive target for developing new adjuvants. In particular, some toll like receptors (TLRs) ligands are either a component of licensed vaccines or are in advanced stages of development [5,6,7]. Signaling through PPRs induces the generation of cytokines, chemokines, and co-stimulatory molecules that activate and polarize immune responses, linking innate and adaptive immunity [8]. 

Adjuvants that activate C-type lectin receptors (CLRs) may be an alternative or complementary to adjuvants targeting TRLs [9]. DC-SIGN, a member of the CLR family, is the subject of increasing interest in the development of vaccines targeting dendritic cells (DCs) [10]. DC-SIGN specifically recognizes carbohydrate structures (high mannose or fucose) on the surface of pathogens. DC-SIGN contains two internalization motifs in its intracellular domain. As a consequence of this structure, upon binding to DC-SIGN, the ligands are internalized through receptor-mediated endocytosis in endosomal and lysosomal compartments, and then processed for major hystocompatibility molecule (MHC)-class I and MHC class II presentation. However some pathogens, including HIV-1, exploit DC-SIGN to escape immunity and promote pathogen infection and dissemination [11]. Interestingly, DC-SIGN activation induces the activation of signal transduction pathways resulting in TLRs signaling, the modulation of immune responses and T cell polarization, with different outcomes depending on the nature of the ligand involved [12]. 

We have recently demonstrated that multivalent pseudo-mannosylated compounds specifically interact with the carbohydrate-recognition domain of DC-SIGN. These glycomimetic compounds inhibit DC-SIGN mediated HIV-1 infection of cellular and tissue models by competing with the binding of the virus to the receptor [13,14,15,16]. In the current work, we selected the most active compound in inhibiting HIV-1 infection and assessed its ability to activate and modulate DC-SIGN signaling. In particular we investigated whether this compound could induce activation of early innate immune responses, with the aim of developing carbohydrate DC-SIGN ligands as adjuvants.

## 2. Results and Discussion

### 2.1.Inhibition of HIV-1 Infection of Human Cervical Tissue by Polyman 19

**Figure 1:**
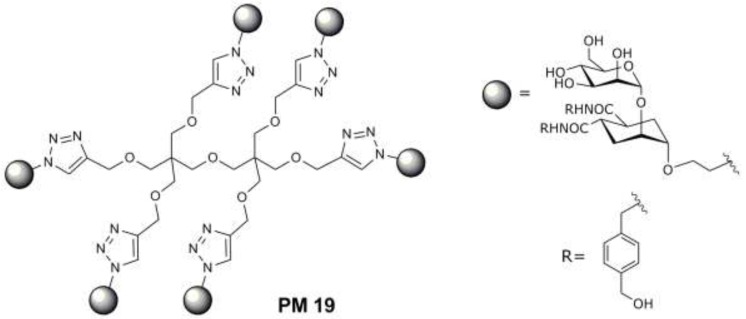
Structure of the hexavalent compound, Polyman 19 (**PM 19**) (on the left side). The detailed structure of the bisamide-based DC-SIGN ligand is reported on the right hand side.

We have recently demonstrated that Polyman 19 (**PM 19**), a polyvalent dendrimer carrying six units of a bisamide-based DC-SIGN ligand (Figure 1), completely inhibits HIV-1 Bal *trans* infection of CD4+ T cells at 10 µM, and reduces the infection by 50% at 1 µM [16]. 

The efficacy of **PM 19** in inhibiting HIV-1 infection was further evaluated exploiting a cervical explant model [14]. Human endocervical tissue was exposed to R5 tropic HIV-1 strains in a non-polarised manner, to simulate a condition of compromised epithelium, and was not activated with IL-2 and PHA, to mimic physiological conditions. 

**Figure 2:**
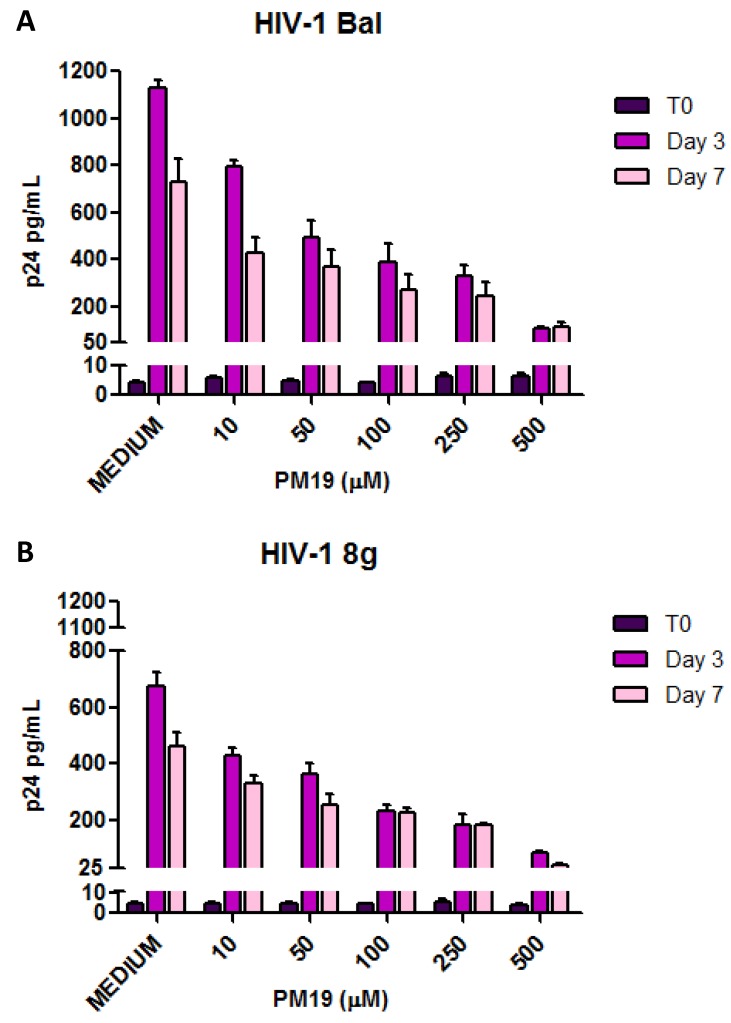
The compound **PM 19** inhibits cervical explants infection by HIV-1 BaL (**A**) and primary isolate 8 g (**B**) in a dose-dependent manner. Experiments were performed on explants from 3 separate donors. Data represent the mean ± SD.

Endocervical explants were pre-treated 30 min with different concentrations of **PM 19** or medium alone before being exposed to HIV-1 BaL or to the HIV-1 clinical isolate 8 g in the continued presence of the compound. After extensive washing to remove unbound **PM 19** and virus, explants were cultured up to 7 days. To verify the removal of unbound virus and quantify the residual inoculum background, the p24 concentration was measured after the washing steps (T0). As shown in Figure 2, the p24 concentration after the washes did not exceed 10 pg/mL for both BaL and 8 g.

Infection was monitored by p24 ELISA at day 3 and 7 post infection. **PM 19** inhibited the infection by both BaL and 8 g in a dose-dependent manner. At the higher concentration assayed (500 µM) **PM 19** diminished the infection by about 90% both at 3 and 7 days post infection (Figure 2). 

Thus, **PM 19** proved to be more effective than other compounds previously tested by our group, such as tetravalent dendron **12**, that needed higher concentration to inhibit CD4 T cells *trans* infection and cervical explant infection [14,16].

### 2.2. *Polyman* **19** Toxicity towards Cervical Explants

**Figure 3:**
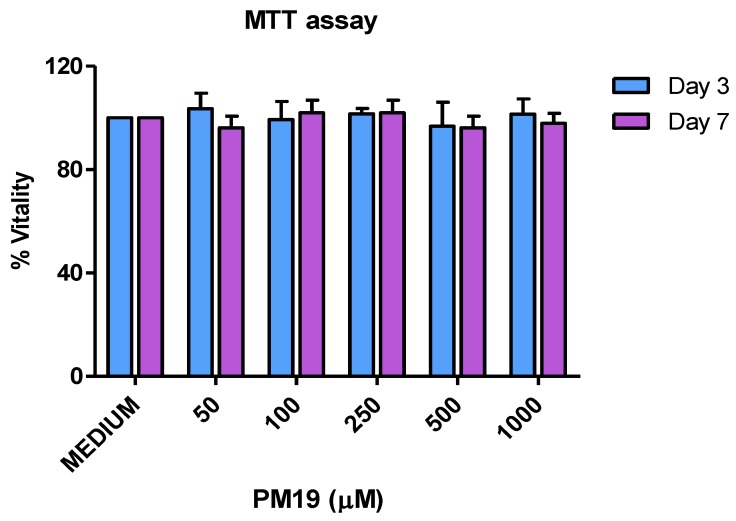
Evaluation of **PM 19** toxicity towards cervical tissue (MTT assay). Experiments were performed on endocervical explants from 3 separate donors. Viability is expressed as percentage of untreated controls. Data represent the mean ± SD.

The effect of the non-polarized exposure of **PM 19** on the viability of cervical explants was estimated by a MTT based assay, in order to determine the potential tissue toxicity of the compound (Figure 3). Endocervical explants were exposed to increasing concentrations of **PM 19** (ranging from 50 to 1,000 µM) for 3 or 7 days. The percentage of viability was calculated dividing the viability of explants treated with **PM 19** (treated) by that of explants exposed to culture medium alone (untreated) . No significant difference in viability between treated and untreated explants was detected up to 1 mM (twice the maximum concentration of **PM 19** tested in the infection experiments).

### 2.3. Modulation of Innate Immune Responses by *Polyman* **19**

Based on result obtained, **PM 19** was chosen to evaluate its capacity to activate innate immune responses. DC-SIGN is expressed by immature Dendritic Cells (iDCs) of derma and mucosal tissues, such as rectum, vagina and uterine cervix. Due to the difficulty of isolating such kind of cells directly from tissues, immature Monocyte-Derived DCs (iMDDCs), that express DC-SIGN and markers of myeloid DCs, were used in the experiments as a model [11].

iMDDCs were differentiated by culturing monocytes obtained from healthy donors in presence of IL-4 and GM-CSF. At day 6 cells were incubated with **PM 19** (100 µM) or medium alone in the presence/absence of LPS or Poly:IC (agonists of TLR4 and TLR3, respectively) for 3 and 8 h. The expression of several genes involved in the early innate immune response and costimulatory molecules was assessed by Real Time PCR. Expression levels were normalized to two house-keeping genes (GAPDH and β-actin), and shown as fold induction compared to untreated control.

**Figure 4:**
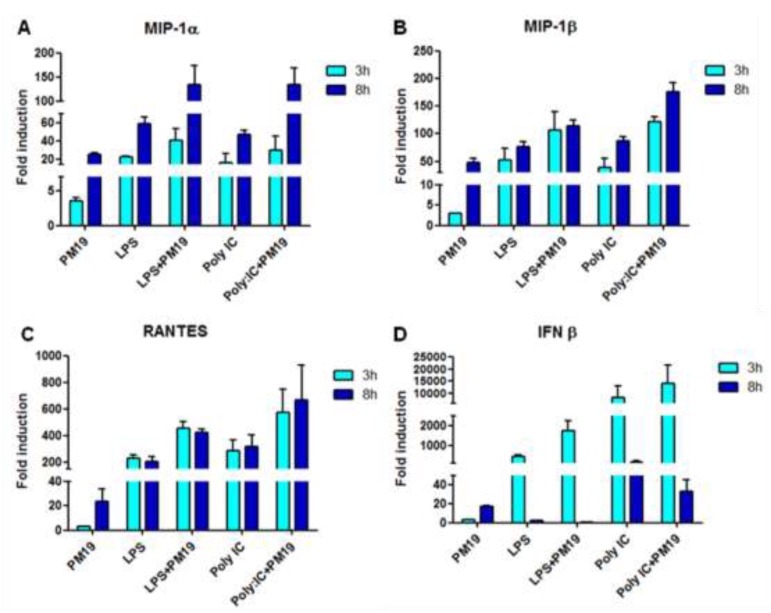
β chemokines (**A**–**C**) and IFNβ (**D**) expression following **PM 19** stimulation for 3 and 8 h. Data are shown as fold change expression from the unstimulated sample. Experiments were conducted on iMDDCs from 3 different healthy donors. Data represent the mean ± SD.

Results indicated that incubation of cells with **PM 19** augments the expression of the β chemokines MIP-1α, MIP-1β and RANTES; this effect is greatly potentiated upon LPS and Poly:IC stimulation, and is evident both at 3 and 8 h (Figure 4A–C). 

MIP-1α, MIP-1β, and RANTES are endogenous ligands of CCR5 and prevent target infection by HIV-1 R5 tropic strains through competitive inhibition [17]. Interestingly, co-delivery of MIP-1α by an adenovirus-based vaccine improved protection in a HIV vaccination mouse model through recruitment and activation of Antigen Presenting Cells (APCs), suggesting its use as adjuvant [18]. 

Beside modulating the production of β chemokines, **PM 19** also augmented the expression of the antiviral cytokine IFNβ both at 3 and 8 h. However this effect was only observed at the 3 h time point when cells were stimulated with either LPS or poly:IC (Figure 4D). 

Notably, **PM 19** did not up-regulate the expression of DC-SIGN itself or of the HIV co-receptors CCR5 and CXCR4 (data not shown).

**Figure 5:**
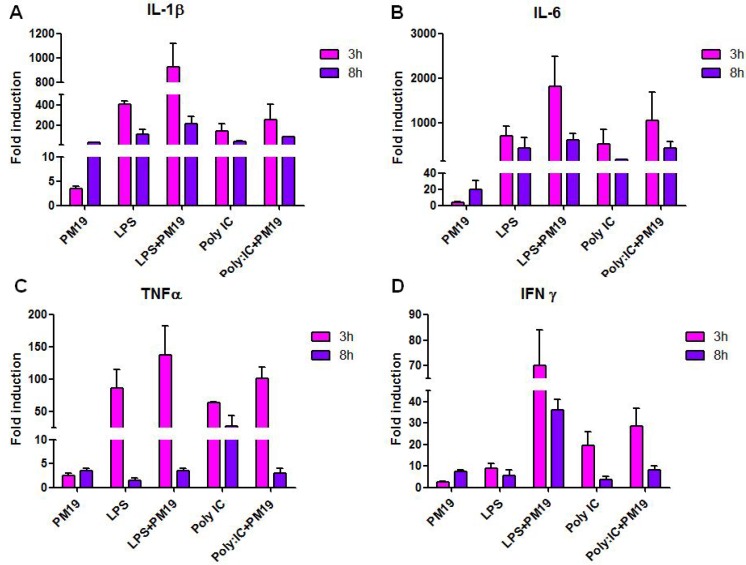
Expression of proinflammatory cytokines IL-1β (**A**), IL-6 (**B**), TNFα (**C**), and IFNγ (**D**) was assessed by Real Time PCR. Experiments were conducted on iMDDCs from 3 healthy donors. Data are shown as fold induction and represent the mean ± SD.

Furthermore, **PM 19** augmented the expression levels of IL-1β, IL-6, TNFα and IFNγ, compared to what observed in untreated controls. Again, this effect was even stronger when **PM 19-**incubated cells were stimulated with LPS or poly:IC. The increased IL-1β, IL-6, and IFNγ expression was observed both at 3 and 8 h, while the effect on TNFα production was seen only at 3 h (Figure 5A–D). 

These results can be biologically important when one considers that INFγ production by DCs and other APCs contributes to early host defenses to viral infections and is involved in autocrine activation and in paracrine activation of nearby cells [19]. 

Proinflammatory cytokines, on the other hand, play an important role in increasing T cell expansion and enhancing CD4+ T cells function. Thus, IL-1β increases antigen-stimulated expansion and differentiation of CD4+ T lymphocytes, and favors persistence of memory cells. Furthermore, IL-1β induces expression of CD40L on CD4+ T lymphocytes and promotes T cell-dependent antibody production in response to protein antigens [20,21]. IL-6 increases CTL responses by down-regulating the expression of the inhibitory molecule PD1 and by inducing Granzyme B production. This cytokine, in association with IL-1, also stimulates CD4+ T cell proliferation and the antigen-stimulated production of IL-2 by these lymphocytes [4,20]. Finally, TNF α is a pleiotropic cytokine produced by several cell types, such as macrophages, DCs, NK cells and activated CD4+ T lymphocytes. TNFα is important for the maturation of DCs and the activation of adaptive immunity following viral infections. In synergy with IL-12 and IL-6, TNFα induces IFNγ expression by both CD4 T+ and CD8+ T lymphocytes, shifting the Th1/Th2 cell balance toward a Th1 phenotype. TNFα also increases the proliferation of CD4+ and CD8+ T cells in response to different stimuli, and enhances antigen specific CTL responses [22,23]. Moreover, it has been recently demonstrated that TNFα induces the production of antigen specific IgG and IgA [23], antibodies that likely play an important role in preventing infection by pathogens that use the mucosa as an entry way [24,25]. 

Interestingly, the compound did not increase the levels of immune suppressive cytokine IL-10 (data not shown), that is associated with reduction of T cells responsiveness and may therefore compromise vaccine efficacy [26,27]. 

**Figure 6:**
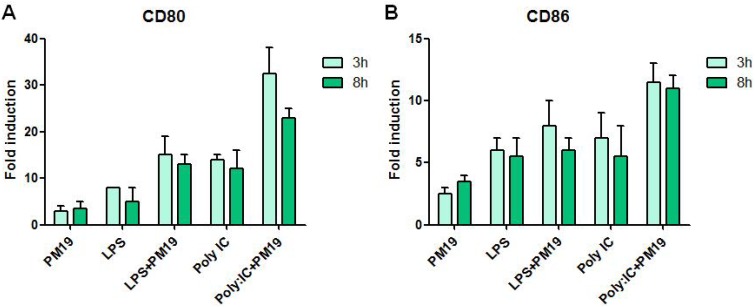
Costimulatory molecules CD80 (**A**) and CD86 (**B**) expression following 3 or 8 h treatment with **PM 19**. Data are shown as fold changes expression from the untreated sample. Experiments were conducted on iMDDCs from 3 healthy donors. Data represent the mean ± SD.

Further analyses showed that incubation with **PM 19** resulted also in the up-regulation of the expression of the important co-stimulatory molecules CD80 and CD86, proteins associated to DC maturation that play a fundamental role in the activation of naïve T cells (Figure 6A,B).

Thus, the hexavalent dendrimer **PM 19** stimulates early immune responses that contribute to counteract HIV infection and activate adaptive immunity. Notably, the compound acts in synergy with TLR agonists such as LPS and Poly:IC. Indeed a combination of adjuvants triggering multiple innate pathways may be more efficient in achieving a protective immune response, especially with difficult vaccine targets such as HIV [6].

In principle, the multivalent structure of **PM 19** allows direct conjugation with antigens, making it possible to realize mixed multivalent dendrimers carrying both DC-SIGN ligands and the desired antigen. This could be a considerable advantage, as it has been demonstrated that co-delivery or covalent conjugation of adjuvant and antigen improve vaccine efficacy [6]. DC-SIGN ligands, after binding to the receptor, are addressed to endo-lysosomal pathway and then presented on MHC II. Furthermore, antigen targeting to DC-SIGN also enhance MHC I presentation, through a cross presentation pathway not yet completely characterized [10]. 

The ability of **PM 19** to trigger multiple beneficial immune responses and to synergize with TLR agonists indicates that the potential usefulness of this novel compound in the construction of vaccines for mucosal pathogens, including HIV-1, should be further investigated.

## 3. Experimental

### 3.1. Human Cervical Explants Infection

HIV-1 BaL (contributed by Drs. S. Gartner, M. Popovic and R. Gallo) was provided through the EU programme EVA centre for AIDS Reagents NIBSC. Primary isolate HIV-1 8 g was provided by Prof. S. Aquaro. 

Endocervical explants (comprising both epithelium and stromal tissue) were obtained from women HIV, HVB, HCV seronegative and without concomitant genital infections, undergoing therapeutic hysterectomy at Sacco Hospital (Milan, Italy), following written informed consent. 

Within 1 h after the surgical procedure, explants were washed, pre-treated with **PM 19** for 30 min, and then exposed 3 h to 200 µL of HIV-1 BaL or 8g (both 2.6 × 10^4^ TCID_50_, corresponding to a gag p24 concentration of 33.4 × 10^3^ and 40.8 × 10^3^ pg/mL respectively) at 37 °C, without washing the compound. Afterward explants were washed five times with RPMI plus 20% Fetal Bovine Serum (FBS) (Euroclone, Siziano, Italy) to remove unbound compound and virus. After the washing, explants were cultured in RPMI medium supplemented with 20% FBS, Penicillin Streptomycin, L-Glutamine (Euroclone) and gentamycin (Sigma-Aldrich, Saint Louis, MO, USA) at 37 °C and 5% CO_2_. Two-thirds of the culture medium was changed at day 3. To measure the residual inoculum background after washing steps (T0), p24 concentration in the last wash was analysed by ELISA. To assess inhibition of HIV infection, supernatants were harvested 3 and 7 days post infection and p24 concentration was assayed. The Alliance HIV-1 p24 Antigen ELISA kit (Perkin Elmer, Waltham, MA, USA) was utilized for p24 quantification. 

### 3.2. Tetrazolium Salt 1-(4,5-Dimethylthiazol-2-yl)-3,5-diphenyl Formazan (MTT) Assay

Toxicity of **PM 19** towards endocervical explants was evaluated by a MTT based assay (*In vitro* toxicology assay, Sigma). Viable tissues reduce MTT dye (yellow) to insoluble purple formazan. Uninfected explants were cultured with increasing concentrations of **PM 19**, or medium culture, for 3 or 7 days. As regards explants treated 7 days, half of the medium culture was changed at day 3, and fresh medium containing **PM19** was added. At the end of the incubation period, the explants were washed with RPMI without phenol red (Euroclone) and incubated in RPMI without phenol red plus 10% FBS and MTT (final concentration 500 µg/mL) for 4 h. Insoluble formazan product was dissolved by a Solubilisation Solution (10% Triton X-100 with 0.1 N HCl in isopropanol). Formazan absorbance was read at 595 nm by a microplate reader (IMark; Biorad, Segrate, Italy). Tissue viability was established by dividing the absorbance reading of the formazan by the dry weight of explants. 

### 3.3. Human Monocyte Isolation

Peripheral blood was collected from buffy coats of healthy donors, following informed consent. Peripheral Blood Mononuclear Cells (PBMC) were isolated by centrifugation on a Ficoll discontinuous density gradient (Lympholyte-H, Cederlane Laboratories). CD14+ monocytes were separated from PBMC by direct magnetic labelling using the CD14 MicroBeads (Miltenyi Biotech), according to manufacturer’s instruction. 

### 3.4. Differentiation of DCs from Monocytes

Purified monocytes (10^6^ cells/mL) were differentiated into iDCs by culturing them in RPMI with 10% Fetal Bovine Serum, Penicillin Streptomycin, L-Glutamine (all from Euroclone) in presence of IL-4 (20 ng/mL) and GM-CSF (20 ng/mL) (both from R&D Systems, Minneapolis, MN, USA) for 6 days. At day 3 the half of culture medium was changed with fresh medium containing IL-4 and GM-CSF.

### 3.5. Flow Cytometry

Differentiation of monocytes to DCs and DC-SIGN expression were confirmed by flow cytometric analysis at day 6. The cells were stained with the listed anti-human monoclonal antibodies: CD11c PE-cy5 (mouse IgG1, clone BU15), CD86 PE (mouse IgG2b, clone HA5.2B7), DRII PE (mouse IgG1, clone Immu357), DC-SIGN PE (clone AZND1), CD83 FITC (mouse IgG2b, clone HB15a), and CD14 PE-cy7 (mouse IgG2a, clone RMO52), all purchased at Beckman Coulter (Cassina De Pecchi, Italy). Labelled cells were analyzed on a CYTOMICS FC-500 flow cytometer interfaced with CXP 21 software (Beckman Coulter). Data were analyzed with Kaloosa software (Beckman Coulter). 

### 3.6. RNA Extraction and Retrotranscription

RNA was extracted using RNA-bee Reagent (Tel-Test Inc, Friendswood, TX, USA) and treated with RNase-free DNase I (New Englands Biolabs, Ipswich, MA, USA) to remove contaminant DNA. cDNA was prepared using random hexamer primers, oligo DT and M-MLV reverse transcriptase (all from Promega, Madison, WI, USA). 

### 3.7. Real Time PCR

cDNA quantification for target genes and the housekeeping genes GAPDH and β actin was performed by the real-time PCR Bioer LineGene 9600 detection system, (CaRli Biotec, Frascati, Italy). Reactions were performed using a SYBR Green PCR mix (Real Mastermix SYBR ROX 2,5 X; 5 PRIME Inc, Gaithersburg, MD, USA). Results were expressed as ΔΔCt and presented as ratio between the target gene and the average of GAPDH and β actin housekeeping mRNA.

## 4. Conclusions

HIV vaccine trials have failed so far; novel ideas are needed (see also [28]). In an attempt to develop new approaches to vaccine design, we developed **PM 19**. Data herein show that this compound inhibits HIV-1 infection of human cervical tissues as a consequence of its tropism for DC-SIGN. 

Moreover, the compound, either alone or in combination with TLR3 and TRL4 agonists, elicits the expression of cytokines, chemokines, and co-stimulatory molecules that are involved in counteracting HIV-1 infection, in activating and recruiting APCs, and in shaping adaptive immunity. Since DC-SIGN is highly expressed by iDCs located in mucosal tissues, a compound interacting specifically with this receptor could be suitable for the use as mucosal adjuvant. 

To further investigate the possible role of **PM 19** (or its derivatives) as an adjuvant, we are realizing dendrimeric structures that include multiple copies of DC-SIGN ligands and selected antigens, aiming at targeting the antigens to mucosal iDCs and enhancing the MHC-I and MHC-II presentation of the linked antigens.
